# Confirmation of emergence of mutations associated with atovaquone-proguanil resistance in unexposed *Plasmodium falciparum *isolates from Africa

**DOI:** 10.1186/1475-2875-5-82

**Published:** 2006-10-04

**Authors:** Christian T Happi, Grace O Gbotosho, Onikepe A Folarin, Danny Milner, Ousmane Sarr, Akintunde Sowunmi, Dennis E Kyle, Wilbur K Milhous, Dyann F Wirth, Ayoade MJ Oduola

**Affiliations:** 1 Malaria Research Laboratories, Institute for Advanced Medical Research and Training, College of Medicine, University of Ibadan, Nigeria; 2 Department of Immunology and Infectious Diseases, Harvard School of Public Health, Boston, MA, USA; 3 Laboratory of Bacteriology and Virology, Dantec Hospital, Dakar, Senegal; 4 Division of Experimental Therapeutics, Walter Reed Army Institute of Research, Silver Springs, MD, USA; 5 Special Programme for Research and Training in Tropical Diseases (WHO/TDR), Geneva, Switzerland

## Abstract

**Background:**

*In vitro *and *in vivo *resistance of *Plasmodium falciparum *to atovaquone or atovaquone-proguanil hydrochloride combination has been associated to two point mutations in the parasite cytochrome b (*cytb*) gene (Tyr268Ser and Tyr268Asn). However, little is known about the prevalence of codon-268 mutations in natural populations of *P. falciparum *without previous exposure to the drug in Africa.

**Methods:**

The prevalence of codon-268 mutations in the *cytb *gene of African *P. falciparum *isolates from Nigeria, Malawi and Senegal, where atovaquone-proguanil has not been introduced for treatment of malaria was assessed. Genotyping of the *cytb *gene in isolates of *P. falciparum *was performed by PCR-restriction fragment length polymorphism and confirmed by sequencing.

**Results:**

295 samples from Nigeria (111), Malawi (91) and Senegal (93) were successfully analyzed for detection of either mutant Tyr268Ser or Tyr268Asn. No case of Ser268 or Asn268 was detected in *cytb *gene of parasites from Malawi or Senegal. However, Asn268 was detected in five out of 111 (4.5%) unexposed *P. falciparum *isolates from Nigeria. In addition, one out of these five mutant Asn268 isolates showed an additional *cytb *mutation leading to a Pro266Thr substitution inside the ubiquinone reduction site.

**Conclusion:**

No Tyr268Ser mutation is found in *cytb *of *P. falciparum *isolates from Nigeria, Malawi or Senegal. This study reports for the first time *cytb *Tyr268Asn mutation in unexposed *P. falciparum *isolates from Nigeria. The emergence in Africa of *P. falciparum *isolates with *cytb *Tyr268Asn mutation is a matter of serious concern. Continuous monitoring of atovaquone-proguanil resistant *P. falciparum *in Africa is warranted for the rational use of this new antimalarial drug, especially in non-immune travelers.

## Background

The rapid development and spread of drug resistant *Plasmodium falciparum *is a serious global health problem in the management of malaria infections. Increasing resistance to antimalarials by *P. falciparum *has led to renewed search for alternative effective new drugs with unique cellular targets. In the 1990s, the urgent need for new anti-malarial drugs for treatment and chemoprophylaxis led to the development of atovaquone (2-[*trans*-4-(4'-chlorophenyl) cyclohexyl]-3-hydroxy-1,4-hydroxynaphtoquinone)[1]. This anti-malarial compound has broad spectrum activity against human protozoan pathogens [2,3] among which are the *Plasmodium *spp. [4,5]. Atovaquone is a potent and specific inhibitor of the cytochrome *bc*1 (*cytbc1*) complex [6,7], an essential respiratory enzyme present in the inner mitochondrial membrane. Unfortunately, in the *Plasmodium *genus and especially in *P. falciparum*, atovaquone when used as a single agent showed a high frequency of recrudescence [8,9] and recrudescing parasites are approximately 1,000–10,000 fold more resistant [10-12]. In order to minimize the risk of resistance development, a fixed synergistic combination of atovaquone with proguanil hydrochloride was developed under the trade name of Malarone^®^. It has been suggested that proguanil at lower doses acts synergistically to enhance the ability of atovaquone to collapse the mitochondrial membrane potential without affecting electron transport inhibition [13]. However, the exact mechanism of synergy between these two drugs remains unknown. The combination of atovaquone-proguanil (AP) can achieve a cure rate of 99%–100% [9,14-16]. Despite its high activity against the malaria parasite, the cost of AP treatment has until now restricted its use to western non-immune adult and children [17] travelers and some European military personnel [18]. Unfortunately, there is growing evidence that malaria parasites may quickly develop resistance to AP by mutation of amino acid residues located in or near the atovaquone-binding site on *cytb *[19-23]. Only a decade after its introduction, AP treatment failures have been reported in non-immune travelers returning from Africa. Nearly all cases are from individuals who have visited West Africa. [11,22,24-27]. AP treatment failures in a significant number of these patients have been genetically linked to point mutations in the atovaquone-binding site of the *P. falciparum *mitochondrial *cytb *gene(Tyr268Ser or Tyr268Asn or Tyr268Cys) [11,12,22,24-28].

Recently, Kessl and colleagues [7] used site directed mutagenesis in *Saccharomyces cerevisiae *to genetically and biochemically confirm the linkage of atovaquone/AP resistance to *cytb *mutations (Tyr268Ser and Tyr268Asn) and to explain at the molecular level the mechanism of malaria parasites resistance to this drug. *Cytb *Tyr268Ser and Tyr268Asn mutations, have been used as a potential molecular marker of AP resistance in non-immune travelers who present with malaria after visiting disease endemic areas [11,12,22,24-30].

It has been suggested that AP resistant phenotypes might arise through strong selection of resistant sub-populations harboring resistance associated mutations [12,29] or, through a mutagenic capacity of atovaquone on *P. falciparum *parasites alone or even in the AP combination [19,20,25] However, very little is known on the background/baseline prevalence of codon-268 mutations in natural populations of *P. falciparum *without previous exposure to the drug in Africa. In this study, the prevalence of codon-268 mutations in the *cytb *gene of *P. falciparum *isolates from Nigeria, Malawi and Senegal was assessed.

Although, no Tyr268Ser mutation is found in *cytb *of *P. falciparum *isolates from Nigeria, Malawi or Senegal, the presence of the *cytb *Tyr268Asn mutation is reported for the first time in unexposed *P. falciparum *isolates from Nigeria. The emergence of *cytb *Tyr268Asn mutation in *P. falciparum *populations of Africa is a matter of serious concern, since the AP combination has not been widely used yet in West Africa.

## Methods

### Study areas

The studies were conducted at the Malaria Research Laboratory, College of Medicine, University of Ibadan, Nigeria (2003–2005), the Queen Elizabeth Central Hospital (within the Blantyre Malaria Project), University of Malawi College of Medicine, Blantyre, Malawi (1996–2005) and the Malaria Research Laboratory, Hopital Le Dantec, Dakar, Senegal (2002–2005).

The Human Subjects Committee of Harvard School of Public Health in Boston, the Institutional Review Committees at the University of Ibadan, Ibadan, Nigeria, the ethical review committee at the College of Medicine, University of Blantyre, Malawi and the Ethics Committee of the Senegalese Ministry of Health approved the protocols used in these studies. Documented informed consent was obtained from parents/guardians.

### Biological materials

Peripheral blood samples preserved on 3 MM Whatman^® ^filter paper were obtained from Nigerian children with microscopically confirmed *P. falciparum*. Parasites DNA samples from Nigerian patients used in this study are the same with those reported in a previous study describing the association between mutations in parasites *dhfr *and *dhps *genes and *in vivo *sulfadoxine-pyrimethamine resistance [31].

Samples from Senegal were collected from patients attending a health clinic in Pikine, a suburb of the capital city, Dakar. A drop of blood obtained from each study participant at enrollment was blotted unto filter paper (ISOCODE, Schleicher & Schuell). The samples were air dried and stored in plastic bags containing silica gel.

In Malawi, parasite DNA was obtained from either an archive of material collected from 1996 to 2004 or as part of an ongoing study of genetic diversity from 2003 to the present. Samples were collected from patients ranging from infants through adult with a spectrum of clinical disease from asymptomatic carriage to severe disease although this information was not used for analysis (i.e., the 91 samples represent the "population" of parasites for Malawi). All samples were either exempt from consent (archived parasite DNA previously extracted and stored at -80°C) or consented for collection of parasite DNA (fresh samples of peripheral blood extracted and stored at -80°C).

### DNA Extraction and PCR-RFLP for detection of codon-268 mutations in the cytb gene

Parasite genomic DNA was extracted from blood samples collected on filter paper using the chelex extraction method as described by Plowe and others[32]. Part of the DNA extracted from each sample was used immediately for PCR and the rest was stored at -20°C. The *P. falciparum *mitochondrial *cytb *gene (GenBank accession no. M99416) was amplified by nested polymerase chain reaction and analyzed by restriction fragment length polymorphisms (RFLP). A nested PCR was designed using *Cytb*1 and *Cytb*2 as primary amplification primers and 3 different pairs of nested primers (*Cytb*2/*Cytb*6, *Cytb*2/*Cytb*7 and *Cytb*3/*Cytb*5) to distinguish the 3 known polymorphisms at codon 268 (Tyr268, Ser268 and Asn268). Primers sequences, primary and nested PCR conditions and procedures were performed as described previously by Schwobel and colleagues [25]. The product of each second round PCR was electrophoresed on 2% agarose gel and visualized under UV transillumination following staining with ethidium bromide.

RLFP analysis of each *cytb *secondary amplification product was performed by digesting 5 μL of each PCR product with the appropriate restriction enzyme and its buffer in a total volume 15 μL and incubated at 37°C over night. The result was detected by electrophoresis in ethidium bromide-stained agarose gel (NuSieve^® ^3:1, Cambrex Bioscience Rockland Inc, ME, USA).

For detection of the wild-type Tyr268 allele at position 268, 5 μL of the secondary amplification product using the primer pair *Cytb*3/*Cytb*5, was mixed with 1 U of the restriction enzyme *NsiI *(New England Biolabs, Beverly, MA). The enzyme cuts the wild-type allele (Tyr268) and mutant Asn268 but not the mutant Ser268 allele. Detection of mutant Ser268 allele was performed by digesting 5 μL of the 171 bp region amplified using the primer pair *Cytb*2/*Cytb*2 with 1 U of the restriction enzyme *AlwNI *(New England Biolabs, Beverly, MA). *AlwNI *cuts the mutant Ser268 allele but not the wild-type Tyr268 or the mutant Asn268 allele. The mutant Asn268 allele was detected by mixing 5 μL of the secondary amplification product (174 bp) using *Cytb*2/*Cytb*7 with 1 U of *SspI *(New England Biolabs, Beverly, MA). The enzyme cuts the wild-type Tyr268 allele or the mutant Ser268 allele but not the mutant Asn268 allele. DNA of *P. falciparum *clones 3D7 (Tyr268 allele), K1 (Tyr268 allele) and NGATV0l (Asn268 allele) strain were used as controls.

### Clonal analysis of PCR products and DNA sequencing

PCR amplified products were cloned into TopoTA^® ^vector (*In vitro*gene, San Diego, CA) and transfected into *E. coli*. Cells were grown overnight in terrific broth and Plasmid DNA was isolated using the SNAP Gel Purification Kit (Invitrogen, Carlsbad, CA). Samples of plasmid DNA containing inserts of interest were purified using a QIAprep Spin Plasmid Kit, and then sequenced using ABI PRISM Big dye Terminator Kit at a commercial facility (Dana Farber Cancer Research Institute, Harvard University, Boston, MA). The *cytb *coding region of mitochondrial DNA from each clone was sequenced five times in both the forward and reverse direction using the vector primers M13 forward and M13 reverse. DNA and protein sequences alignment was performed using the SeqMan™ and MegAlign™ (Seqwright, Houston, TX; DNASTAR, Madison WI) and MUSCLE [33] protein multiple sequence alignment softwares. *P. falciparum *clones 3D7 (Tyr268 allele), K1 (Tyr268 allele) and NGATV0l (Asn268 allele) strain were used as controls.

## Results

A total of 309 samples were obtained from patients in Nigeria (118), Malawi (93) and Senegal (98). Out these samples, 295 (Nigeria 111; Malawi 91 and Senegal 93) were successfully tested for two different mutations on codon 268 of the parasite's *cytb *gene, which has been associated previously with AP treatment failure [12,20,22-27]. Seven (7), 2 and 3 patient samples from Nigeria, Malawi and Senegal respectively did not yield PCR products. Patients characteristics are presented in Table 1. All patients were Africans resident of malaria endemic areas of West Africa (Nigeria and Senegal) and East Africa (Malawi) where AP is neither used for chemoprophylaxis nor for treatment of malaria.

**Table 1:** Characteristics of patients from whom samples were used for detection of *cytb *mutations at codon 268.

**Country**	**Number of patients**	**Age range (years)**
**Nigeria**	111	1–12
**Malawi**	91	0.5–25
**Senegal**	93	3–70

In the parasite populations studied, PCR-RFLP results revealed no *P. falciparum *isolate from Nigeria, Senegal or Malawi with the mutant Ser268 allele in the *cytb *gene. All isolates from Senegal and Malawi harboured the wild-type *cytb *Tyr268 allele. In addition, none of the isolates obtained from these 2 study sites showed the mutant *cytb *Asn268 allele.

**Figure 1:**
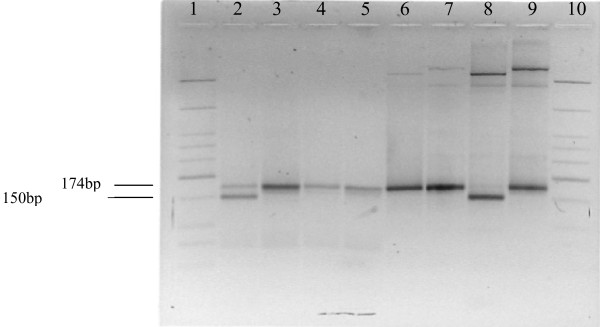
Detection of Asn268 mutation in cytochrome b gene in *P. falciparum *by Restriction digest method. 174 bp amplification products with the secondary amplification primer pair *cytb*2/*cytb*7 (lanes 3, 5, 7, 9) digested with SspI (lanes 2, 4, 6, 8) and were run on 2% NuSieve^® ^3:1 agarose gel. DNA from NGATV01 containing the AAT (Asn) mutation remains uncut (lane 6), while DNA from K1 containing the TAT (Tyr) wild-type codon is digested (150 bp) by the enzyme (lane 8). DNA from patient ID011 showed a mixed infection consisting both the TAT (Tyr) wild-type digested (150 bp) and mutant undigested (174 bp) codons (lane 2). Patient ID024 had parasites harboring the mutant (Asn268) allele of *cytb *as their DNA remained uncut (lane 4) by the enzyme. Lanes 1 and 10 represent the low molecular weight DNA ladder (New England Biolabs, Beverly, MA) used as a marker for the electrophoresis

**Figure 2:**
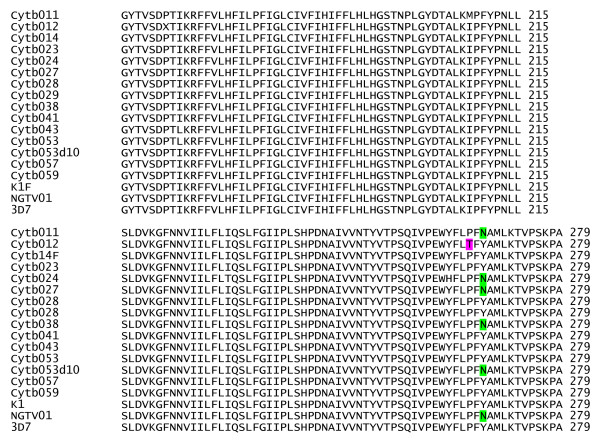
Multiple sequences alignment of cytochrome b gene (residues 137 to 279) of some Nigerian isolates of *P. falciparum*. Highlighted are residues 268 with amino acid changes from the wild-type tyrosine (Y) to the mutant asparagine (N) allele associated previously with atovaquone resistance in *Plasmodium falciparum*. In addition residue 266 in patient ID (Cytb012) where Pro (P) is changed to Thr (T) is also highlighted. Sequences of the atovaquone resistant (NGTV01) and sensitive (K1 and 3D7) control strains are also present.

Surprisingly, five isolates out of the 111 samples obtained from Nigerian patients showed the mutant *cytb *Asn268 allele. The resulting pattern after restriction enzyme digest of some of these five samples is shown in Figure 1. SspI cut the 174 bp product of the nested PCR into two fragments (150 bp and 24 bp) in presence of the TAT (Tyr) wild-type codon, while the mutant AAT (Asn) codon remained uncut. Fragment sizes between the wild-type *cytb *Tyr268 and mutant *cytb *Asn268 differed by 24 bp (Figure 1). One (1) out of the five (5) samples that revealed the mutant Asn 268, showed a mixed (Tyr268 and Asn268) pattern, consisting of wild-type and mutant alleles at codon 268. The presence of these mutations was further confirmed by sequencing. Sequencing data (Figure 2) confirmed the presence of the Asn268 mutation in all the five samples from which the mutant alleles were detected by RFLP.

## Discussion

In this study, the background/baseline prevalence of AP associated resistance mutations in *P. falciparum cytb *gene in isolates from three distinct geographical areas of Africa where the drug has not been introduced for the treatment of *P. falciparum *malaria was determined. The data from the study provides the very first evidence of the presence of *cytb *Tyr268Asn mutation in unexposed *P. falciparum *isolates from Africa.

In 2002, the first case of *in vivo *resistance to AP was reported in a non-immune European traveler returning from Nigeria [22]. Molecular and phenotypic characterizations of the isolate of *P. falciparum *obtained from the patient showed a Tyr to Asn mutation at codon 268, resulting in a 800 fold increase of the IC_50 _[22]. However, Fivelman and colleagues [22] argued that since AP combination is not yet used in West Africa, it is unlikely that the patient was initially infected with an atovaquone resistant strain of *P. falciparum*. Thus, the mutation in the parasites *cytb *gene would have occurred under AP pressure. The data from this study is contradictory to the argument raised by Fivelman and colleagues [22], since AP combination is still not used in Nigeria and yet some *P. falciparum *from the same locality harbor mutant Asn268 allele.

The reasons behind the emergence of the mutant Asn268 allele of *cytb *gene is indigenous *P. falciparum *unexposed to AP combination is remain unclear. It has been suggested that the mutations at codon 268 of *cytb *gene in unexposed *P. falciparum *populations may occur either naturally [11] or through prior exposure to related drugs [27]. Trimethoprim-sulfametoxazole is often used for treatment of *Pneumocystis carinii *pneumonia in HIV patients in Nigeria. However, for patients who react to this sulfa-based combination, atovaquone is given as a rescue therapy. It is possible that treatment with atovaquone in this category of patients could select for mutant Asn *cytb*268 in indigenous *P. falciparum *population in this area where HIV patients could be co-infected with malaria, although there is no clear evidence of this selection process. A recent report [34] has shown that the percentage of individuals with *Pneumocystis carinii *pneumonia who are co-infected with other pathogens is very high (ranging from 20% to 70%) in Africa and other developing countries.

Another potential explanation for the emergence of *P. falciparum *with *cytb *Asn268 mutation in Nigeria could be due to the selection of this allele by AP in the large number non-immune (European and Americans) workers (under AP chemoprophylaxis) in the Nigerian oil industry. Investigations are currently underway to determine if AP chemoprophylaxis among non-immune oil workers is exerting some selective pressure on indigenous strains of *P. falciparum*.

The detection and confirmation of mutant *cytb *Asn268 allele in some *P. falciparum *isolates from Nigeria is a matter of serious concern since these parasites may spread to other neighboring African countries where *P. falciparum *resistance to atovaquone or AP combination has not been reported. Recent studies [7,12,22,23] confirmed genetically and biochemically the linkage of atovaquone/AP resistance to *cytb *mutations (Tyr268Ser and Tyr268Asn) and the molecular mechanisms of parasites resistance this drugs. Tyr268 is a conserved bulky hydrophobic contact of atovaquone in the Qo II region of the ubiquinol oxidation site of the *cytb*c1 complex. Substitution of Tyr268 by a less bulky Asn268 not only reduces the volume of the binding pocket, but also decreases the affinity and binding of atovaquone and thus leads to drug resistance [22,35]. Of interest, is the presence in one sample (Cytb012) of an addition mutation at position 266, where Pro (P) is substituted to Thr (T). The presence and the role of this mutation within the atovaquone binding site needs further investigation, as the presence of additional mutations within the ubiquinone reduction site has been previously suggested to be involved in high level resistance to the drug [7].

Efforts are currently underway to conduct a more elaborate study where genotypic and phenotypic characterization of AP resistant isolates of *P. falciparum *will be determined especially in Nigeria.

## Conclusion

This study reports for the first time the presence of Tyr268Asn mutation in unexposed *P. falciparum *isolates from Nigeria. The emergence in African *P. falciparum *isolates of Tyr268Asn mutation which is associated with AP resistance raises serious concerns about the long-term use of this drug for malaria chemoprophylaxis in non-immune travelers visiting West Africa. Continuous monitoring of AP resistant *P. falciparum *in Africa is warranted for the rational use of this valuable antimalarial drug especially in non-immune travelers.

## Authors' contributions

HCT, GOG, OAF, AS, KED, MKW, WFD and AMJO contributed to the conception and design of the study. HCT, GOG, OAF, MD, OS and AS participated in enrolment of patients and sampling. HCT, GOG, OAF, MD, OS performed molecular typing of isolates and analysis of data. All the authors contributed to writing the manuscript. All the authors read and approved the final version that was submitted for publication.

## Disclaimer

The opinions or assertions contained herein are the private views of the authors (MKW and KED), and are not to be construed as official, or as reflecting true views of the Department of the Army or the Department of Defense.
